# Single-cell transcriptional profiling reveals the heterogenicity in colorectal cancer

**DOI:** 10.1097/MD.0000000000016916

**Published:** 2019-08-23

**Authors:** Weier Dai, Fangbin Zhou, Donge Tang, Liewen Lin, Chang Zou, Wenyong Tan, Yong Dai

**Affiliations:** a College of Natural Sciences, The University of Texas at Austin, Austin, TX; b Clinical Medical Research Center, The Second Clinical Medical College of Jinan University (Shenzhen People's Hospital), Shenzhen; c Integrated Chinese and Western Medicine Postdoctoral Research Station, Jinan University, Guangzhou; d Department of Oncology, Shenzhen Hospital of Southern Medical University, Shenzhen, China.

**Keywords:** cell markers, cluster, colorectal cancer, heterogenicity, single-cell RNA-sequencing

## Abstract

**Background:**

Colorectal Cancer (CRC) is a highly heterogeneous disease. RNA profiles of bulk tumors have enabled transcriptional classification of CRC. However, such ways of sequencing can only target a cell colony and obscure the signatures of distinct cell populations. Alternatively, single-cell RNA sequencing (scRNA-seq), which can provide unbiased analysis of all cell types, opens the possibility to map cellular heterogeneity of CRC unbiasedly.

**Methods:**

In this study, we utilized scRNA-seq to profile cells from cancer tissue of a CRC patient. Gene ontology (GO) and Kyoto Encyclopedia of Genes and Genomes (KEGG) pathway analyses were performed to understand the roles of genes within the clusters.

**Results and Conclusion:**

The 2824 cells were analyzed and categorized into 5 distinct clusters by scRNA-seq. For every cluster, specific cell markers can be applied, indicating each 1 of them different from another. We discovered that the tumor of CRC displayed a clear sign of heterogenicity, while genes within each cluster serve different functions. GO term analysis also stated that different cluster's relatedness towards the tumor of CRC differs. Three clusters participate in peripheral works in cells, including, energy transport, extracellular matrix generation, etc; Genes in other 2 clusters participate more in immunology processes. Lastly, trajectory plot analysis also supports the viewpoint, in that some clusters present in different states and pseudo-time, while others present in a single state or pseudo time. Our analysis provides more insight into the heterogeneity of CRC, which can provide assistance to further researches on this topic.

## Abstract 2

Supplemental Digital Content is available in the text

## 1 Introduction

Tumor heterogeneity poses a serious challenge to cancer treatment and patient survival. It can be divided into inter-tumoral and intra-tumoral heterogeneity. The former is observed that variability occur between patients with the same histologic type, and forms the cornerstone of targeted cancer medicine.^[1,2]^ The latter is further present within a single tumor. Cells from the same tumor may harbor different genetic mutations or exhibit distinct phenotypic states.^[3–5]^ Such intra-tumoral heterogeneity is increasingly appreciated as a key determinant of treatment failure, drug resistance, and disease recurrence.^[2]^ It is therefore indispensable to fully characterize the phenotypes and interactions of diverse cell types present within tumors.

Bulk cell RNA-sequencing (RNA-Seq) technology has been widely used for transcriptome profiling to study transcriptional structures, splicing patterns, and gene and transcript expression levels.^[6,7]^ However, such ways of sequencing can only target a cell colony and obscure the signatures of distinct cell populations. Alternatively, recently developed single-cell RNA sequencing (scRNA-seq) technologies provide comprehensive, unbiased analysis of all cell types and states based on gene activity within tumor masses.^[8,9]^ It opens the possibility to map cellular heterogeneity unbiasedly, recovers cellular identities independently of a priori defined labeling strategies and can furthermore uncover either their previously unrecognized disease-associated cell populations or functional states, novel biomarkers and potential molecular regulators.^[10]^ Multiple studies have used scRNA-seq to investigate the intra-tumoral heterogeneity in several cancer types.^[11,12]^ Patel et al used scRNA-seq to profile 430 cells from 5 primary glioblastomas, and found inherently variable in their expression of diverse transcriptional programs related to oncogenic signaling, proliferation, complement/immune response and hypoxia.^[11]^ Min et al analyzed transcriptomic data obtained from 34 single cells from human lung adenocarcinoma (LADC) patient-derived xenografts (PDXs) and revealed 2 distinct intra-tumoral subgroups that were primarily distinguished by the gene module G64.^[12]^ The above results indicate that scRNA-seq may have the ability to characterize the phenotypes and interactions of diverse cell types present within tumors.

Colorectal cancer (CRC) is the third most common cancer in the world, and about 1.4 million cases were diagnosed worldwide in 2012.^[13]^ It was characterized by heterogeneous features of histopathologic, genomic, epigenomic, and transcriptomic alteration. Bulk transcriptomic analysis shows that stromal cell signatures correlate with risk of recurrence and predict patient survival, highlighting the importance of diverse cell populations in CRC.^[14,15]^ In this study, scRNA-seq was performed to generate a molecular census of cell types and stated with the cancer tissue from a CRC patient. Combining this technology with clustering analysis to characterize gene expression at the single cell level and resolve differential responses by cell type can help us understand how activated and quiescent, abnormal cellular subpopulations contribute to CRC disease initiation, maintenance, and progression.

## 2 Methods

### 2.1 Study subject

This study involved a 73-years old female Stage III right Colorectal cancer patient. Clear pathologic diagnosis and clinical evidence were confirming as CRC. Cancer tissue was dissociated from the patient and used for scRNA-seq analysis. This study was conducted by the Declaration of Helsinki and was approved by the Ethics Committee of the Shenzhen People's Hospital, China (Ref No. 2015-313); the sole participant also provided her written informed consent.

### 2.2 Dissociation of tumor

To ensure accurate and satisfactory results, fresh tissue was required for scRNA-sequencing. Cancer tissue from the CRC patient was obtained and immediately dissociated by Tumor Dissociation Kit, human“” (Miltenyi Biotec, Germany) as the vendor recommended. In detail, an enzyme mix was firstly prepared by combing various types of enzymes. Then, the cut 2 to 4 mm tumor was added to a gentle MACS C Tube along with the enzyme mix. The tube was attached upside down to the genleMACS Dissociator and process by the gentleMACS Program. Afterward, the C Tube was detached and incubated for 30 minutes at 37°C while rotating continuously using the MACSmix Tube Rotator. Later, the sample was resuspended and applied to a MACS∗ SmartStrainer placed on a 50 mL tube. The cell was washed with 20 mL of RPMI 1640. Lastly, the cell was centrifuged at 300 × *g* for 7 minutes. Both the cancer tissue and adjacent tissue were prepared and used for sc-RNA sequencing analysis.

### 2.3 Single-cell capture, library preparation, and RNA-seq

The cell suspension was diluted to a concentration of 11 × 0^6^ cells/ml in PBS plus 0.04% bovine serum albumin (BSA). The cell viability was at least 80%. Cell count and viability were determined using trypan blue on a Countess FL II. Then, scRNA-seq libraries were prepared according to the Single Cell 3′Reagent Kit User Guide v2 (10× Genomics).^[16]^ Briefly, cellular suspensions were loaded on a Chromium Controller instrument (10× Genomics) to generate single-cell gel bead-in-emulsions (GEMs). GEM-reverse transcriptions (GEM-RTs) were performed in 96-deep well reaction module: 55°C for 45 minutes, 85°C for 5 minutes; end at 4°C. After the RT step, GEMs were broken, and barcoded-cDNA was purified with DynaBeads MyOne Silane Beads (Thermo Fisher Scientific, 37002D). Subsequently, cDNAs were amplified with 96-Deep Well Reaction Module (98°C for 3 minutes; cycled 12 times: 98°C for 15 second, 67°C for 20 second, and 72°C for 1 minute; 72°C for 1 minute; end at 4°C) and cleaned up with the SPRIselect Reagent Kit (Beckman Coulter, USA). Indexed sequencing libraries were constructed using the GemCode Single-Cell 3′ Library Kit for enzymatic fragmentation, end-repair, A-tailing, adaptor ligation, ligation cleanup, sample index PCR, and PCR cleanup.^[16]^ Quantification of the constructed libraries was evaluated using QubitdsDNA HS Assay Kit (Thermo Fisher), Agilent cDNA High Sensitivity Kit, and Kapa DNA Quantification Kit for Illumina platforms, following the manufacturer's instructions. The library was sequenced on Illumina HiSeq2500, using the paired-end 2 × 125 bp sequencing protocol. Sequencing run parameters were set up according to version 2 chemistry, the number of cycles for each read as follows: Read 1: 26 cycles, i7 index: 8 cycles, i5 index: 0 cycles and Read 2: 98 cycles.^[17]^

### 2.4 Processing of sc-RNAseq data

CellRanger software was utilized to analyze Single-cell expression (version 2.1.1); it was also used to perform quality control, sample de-multiplexing, barcode processing, and single-cell 3’gene counting. Sequencing reads were aligned to the UCSC hg38 transcriptome using the default parameters in Cell Ranger suite. For Quality Control (QC), we filtered out genes detected in less than 3 cells and cells where < 200 genes had nonzero counts. Also, we removed cells having a percentage of total unique molecular identifiers (UMIs) that derived from mitochondrial genome greater than 10%, as such data may result from conditions such as cells undergoing stress and cell death. Filtered data were normalized and log-transformed; later, the log-transformed matrix was used for all downstream analysis. The cluster identities defined by K-means clustering in Cell Ranger Based on Louvain Modularity optimization algorithm. DDRTree method was adopted for pseudo time analysis; generally, genes with the top 1500 highest standard deviations were obtained as highly variable genes. These genes were used for dimensionality reduction utilizing Principle component analysis (PCA). Using the first 10 principal components as input, t-distributed stochastic neighbor embedding (t-SNE) was used for data visualization in 2 dimensions.

### 2.5 Cluster cell type annotation and marker genes identification

We used the feature plot function to highlight the expression of known marker genes to identify clusters. Violin plots for the given genes were generated using the Seurat toolkit VlnPlot function. Also, Gene ontologies of DEGs were assessed using the PANTHER Overrepresentation Test (Gene Ontology Consortium, http://www.geneontology.org/), with the reported *P* values corrected by the Bonferroni adjustment for multiple comparisons.

### 2.6 Statistical analysis

Major cell type-specific markers were screened by Seurat's Bimod likelihood ratio statistical test, satisfying *P*-value ≤.05 and greater than or equal to 2 times the differential expression range screens the differential gene between the specified cell population and the remaining cell population. These markers are further subjected to bidirectional hierarchical clustering of genes and samples. According to log2FC, A heat map of the top 10 genes that were unique to each cluster was displayed.

## 3 Results

**Figure 1:**
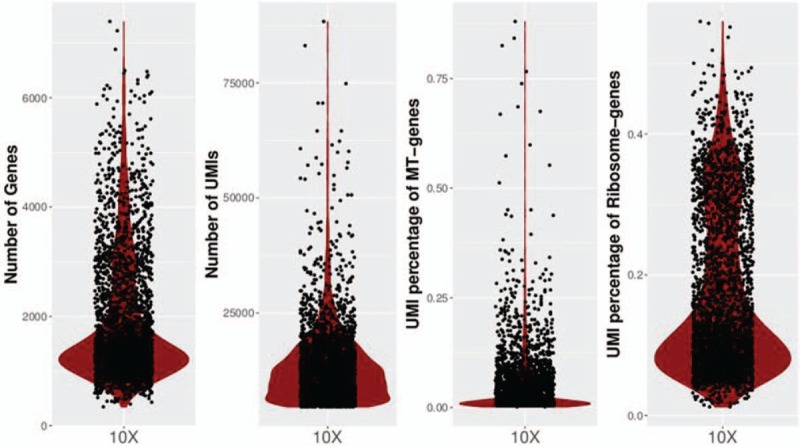
Violin plot of number of genes, UMIS, and the percentage of UMIs that derived from mitochondrial and ribosomal genome detected per single cell for cancer tissue. UMIs = unique molecular identifiers.

A deep transcriptional map of cancer cell states and gene expression in CRC patient at single-cell level was generated after the performance of scRNA-seq on cancer tissue from a CRC patient. The 2824 cells were obtained for further analysis. The median number of UMIs and genes detected per cell were 11,731 and 21,111. 69.6% of sequencing saturation rate indicated comprehensive sampling of the available transcripts. Above data can be seen from Figure 1.

### 3.1 Cell markers applying to each cluster

**Figure 2:**
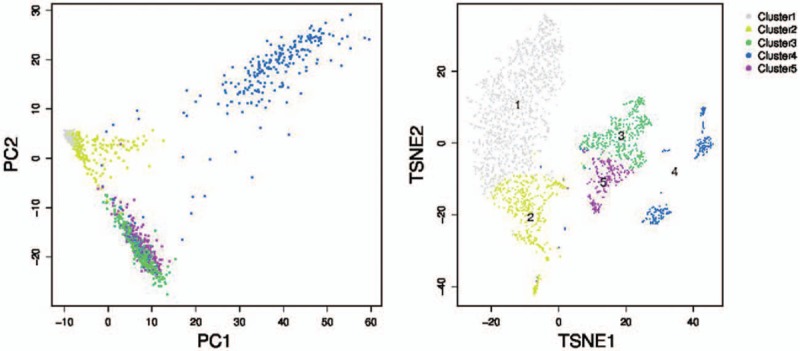
Cell ranger analysis and monocle analysis reveals the distribution of 5 clusters.

**Figure 3:**
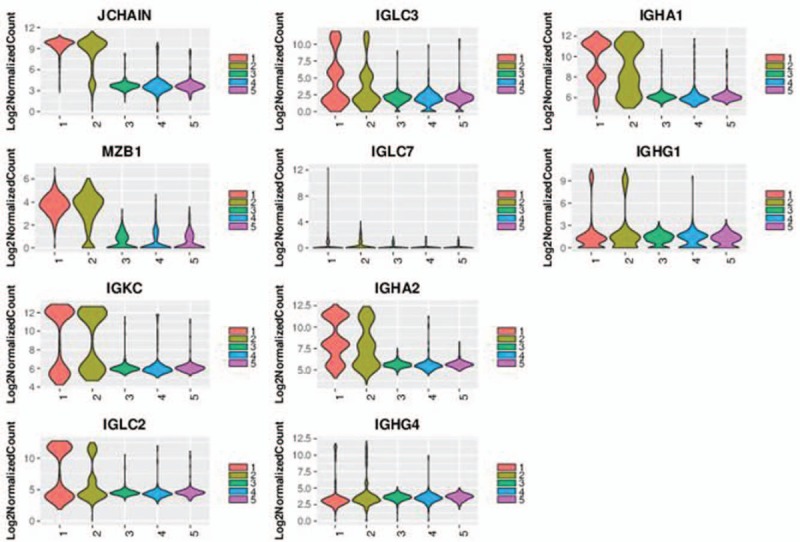
Violin plot indicating the expression distribution of the top 10 marker genes of Cluster 1.

A total of 5 clusters were recognized after scRNA-seq (Fig. 2). Upon analyzing specific genes in each cluster, cell markers can be placed to each one of them. After careful research, it was determined that cluster 1 mainly consists of immune cells, since IGL@ and IGH@ both recognize foreign antigens and initiate immune responses (Fig. 3). cluster 2 contains specific genes related to the major histocompatibility complex (see figure, Supplemental Digital Content 1, which indicates the expression distribution of the top 10 marker genes of cluster 2); cluster 3 mainly consists of genes serving to stabilize the cell, energy transportation and cell regulation such as, TSPAN6, PFDN4, and TIMM13 (see figure, Supplemental Digital Content 2, which indicates the expression distribution of the top 10 marker genes of cluster 3); cluster 4 majored in breakdown of extracellular matrix and remodeling of tissues (see figure, Supplemental Digital Content 3, which indicates the expression distribution of the top 10 marker genes of cluster 4); and cluster 5 contain genes that will express themselves in cancer, such as WFDC2 (see figure, Supplemental Digital Content 4, which indicates the expression distribution of the top 10 marker genes of cluster 5). Upon placing markers on each cluster, further analysis can be more synthesized and clearer.

### 3.2 Major cell type-specific markers

**Figure 4:**
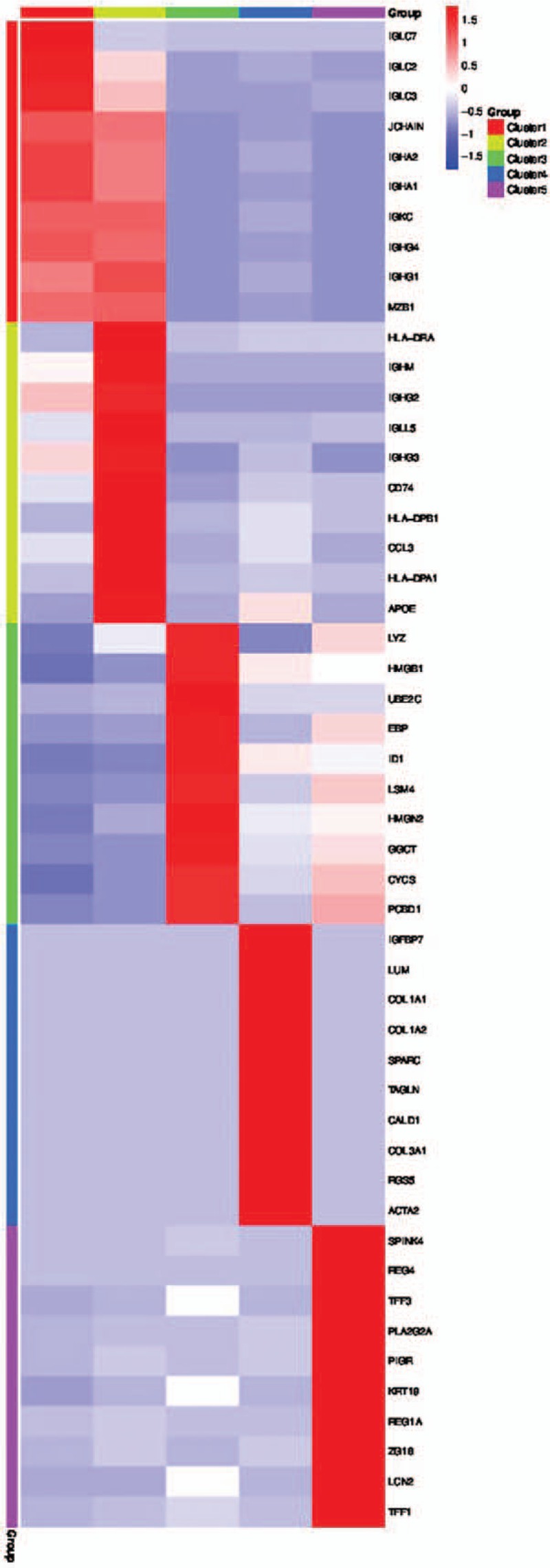
Heatmap of the top 10 marker gene per cluster.

To further differentiate between each cluster, analysis were performed on differentially expressed genes between cell types based on mean expression and covariance patterns; we discovered marker gene sets were sufficient to identify cell type with high probability uniquely. A heat map of the top 10 genes that were unique to each cluster based on average log fold-change showed a high degree of heterogeneity between the clusters (Fig. 4). Cluster 1 was distinguished by having a unique set of genes such as IGLC7, IGLC2, IGLC3. Cluster 2 possesses unique genes such as HLA-DRA, IGHM, IGHG2. Other 3 clusters also define themselves with unique genes as well. It can also be witnessed that these top 10 genes unique to each cluster have different UMI counts. Moreover, the top 10 most distinct signature genes in each cell type were presented as violin plots and compared for the differences between each cluster within the Cancer tissue.

### 3.3 Differential expression genes-GO/KEGG analysis

**Figure 5:**
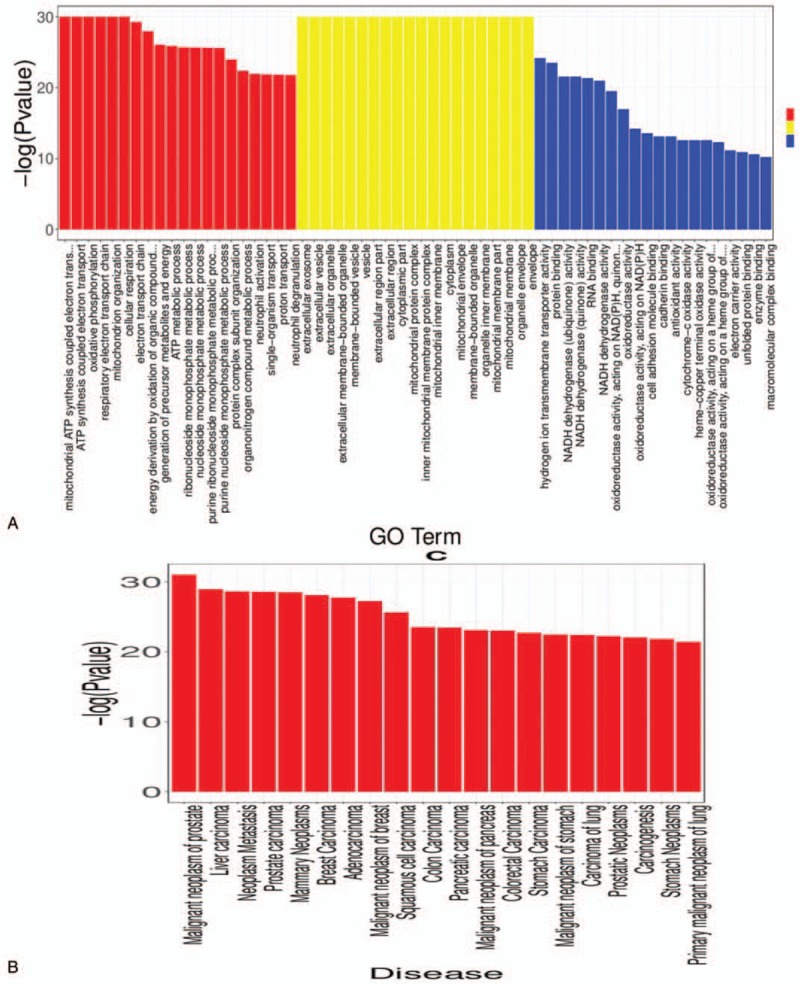
(A) GO analysis of the selected top 10 GO terms for cluster 1; (B) Disease enrichment plot from KEGG pathway analysis for cluster 1. GO = Gene ontology, KEGG = Kyoto Encyclopedia of Genes and Genomes.

To further investigate CRC disease-associated cell functional states and potential molecular regulators, gene ontology (GO) analysis of differentially expressed genes (DEG) in cancer tissue. Cluster 1 revealed a higher proportion of biological processes genes, including, ATP synthesis, cellular respiration, oxidative phosphorylation, and mitochondrion organization (Fig. 5). Cluster 2, also abundant in biological process genes, consist of GO-terms such as cell activation, positive regulation, response to stress, cellular response, and cell adhesion (see figure, Supplemental Digital Content 5, which indicates GO analysis and disease enrichment plot for cluster 2). Cluster 3 has a set up comparable to Cluster 1; by which, it also has a high biological processes gene proportion, and the primary function reveals to be similar (see figure, Supplemental Digital Content 6, which indicates GO analysis and disease enrichment plot for cluster 3). Cluster 4 possesses a high percentage of biological processes which is responsible for extracellular matrix organization, response to stress, locomotion, cell migration, and cell motility (see figure, Supplemental Digital Content 7, which indicates GO analysis and disease enrichment plot for cluster 4). Lastly, Cluster 5 has a similar set up as Cluster 2; mostly biological processes and similar main functions (see figure, Supplemental Digital Content 8, which indicates GO analysis and disease enrichment plot for cluster 5). KEGG pathway analysis also demonstrated disease enrichment in each cluster. It revealed that cancer-related disease present in all 5 clusters, such as neoplasm metastasis, colorectal cancer, malignant neoplasm of breast, etc; This confirmed the reliability of the experimental results and indicated that CRC is a highly complex disease.

### 3.4 Cell states displayed heterogeneity in CRC

**Figure 6:**
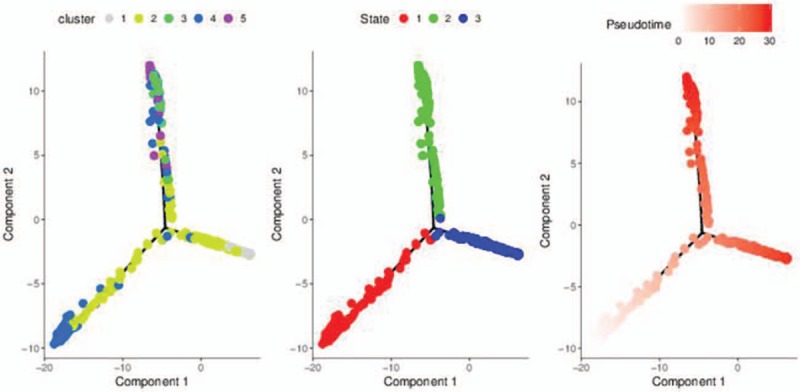
Trajectory plots differentiated by clusters, states, and pseudotime.

We generated trajectory plots to determine the relatedness of cells types and states. Then, several trajectories were constructed in pseudo temporal order; this can provide us with a clear view of both branched and linear differentiation. Theoretically, cells resided on the same or adjacent branches are believed to be more hierarchically related than cells on neighboring branches in a specific trajectory plot. Thus, it was revealed that there were 5 distinct cell clusters in cancer tissue. As shown in Figure 6, cluster 2 to 5 presents in state 1; cluster 1, 2, and 4 presents in state 2; and cluster 2, 4, and 5 presents in state 3. It revealed that some clusters overlapped in different states.

## 4 Discussion

CRC is one of the most concerned types of cancer in the world. Pathogenesis is still not clearly understood; many believe genetic and environmental factors contribute to this disease. However, the role of each type of signaling molecules and immune cells plays in this disease remains obscure. Sc-RNAseq is the new technology that can prevent aberrant signals from overlooking and provide more insight into cells itself for more synthesized observation and analysis.^[18]^ Here, we report comprehensive single-cell expression profiling of cancer tissue of CRC patient using this technology. The 2824 cells were obtained and further classified into 5 distinct cell clusters whereas each cluster was made up of a different number of cells. We suspect that the tumor of CRC may display heterogeneity. This point of view becomes more evident as the analysis went on. Initially, the analysis revealed that cell markers can be applied to each cluster; for example, Cluster 2 mainly consist genes related to Major Histocompatibility Complex (MHC), while the remaining 4 clusters possess cell markers uniquely to themselves. Also, the generated heatmap displayed heterogeneity between each cluster, due to each distinct cluster defined themselves with a unique set of genes; This supported the hypothesis of heterogeneity in the tumor of CRC.

Another evidence that supports the above viewpoint was the results from GO term analysis and KEGG pathway analysis. GO term analysis demonstrates that each cluster serves different functions. GO term analysis of cluster 1 revealed that there were genes responsible for biological processes including, ATP synthesis, cellular respiration, energy derivation and so on; other genes are cellular components; remaining part of those genes were responsible for molecular function, serving to bind protein, RNA, dehydrogenate NADH, adhesion of cells. Other 4 clusters that underwent GO term analysis indicated similar pattern whereas genes in the same cluster served different functions. Moreover, GO term analysis demonstrated the relatedness towards the tumor differs for each cluster. Apart from Cluster 1 written above, Cluster 3 and 4's genes mainly served to support cells (providing energy, generating extracellular matrix) Genes of cluster 2 and 5 emphasized more on immunity; functions included immune response, regulation of lymphocyte, leukocyte, T-cell activation and so on indicating these 2 clusters were more related to the tumor. However, KEGG pathway analysis of disease enrichment revealed that carcinoma diseases presented in all 5 clusters. These discoveries proved that, apart from diversities of genes within a cluster, each cluster's relatedness to the tumor also differs. Again, such pieces of evidence enforced the idea that tumor of CRC displayed numerous heterogeneity.

Trajectory plot analysis was another piece of evidence demonstrating CRC's heterogeneity. As stated above, trajectory plots were analyzed based on clusters, states, and pseudo-time. Results showed several clusters were present in the same state, while a specific cluster spans over several states. Also, clusters were observed overlapping in several pseudo time regions. For example, Cluster 2 exists in all 3 states; Cluster 1, 2, and 4 all present in state 3; This lead to the conclusion that tumor of CRC was indeed heterogeneous, and different clusters participated disproportionally within a tumor.

In conclusion, the sc-RNAseq of cancer tissue from CRC patient allowed for unbiased, readjusting identification of distinct cell types and states and can provide us with a detailed insight. By utilizing this technology, analysis towards clusters, GO term, KEGG and Trajectory plot supported the viewpoint that CRC is indeed heterogeneous in numerous aspects. During the research, it was somehow regrettable that only one case of Stage III CRC can be collected. In addition, further analysis such as qPCR was not able to perform. Combining further analysis with comparison between the tumor and cancer peripheral tissue will probably yield more promising evidence. Thought the fact, existing data can still present a comprehensive view and insight towards CRC in a whole; others can utilize the data to develop more strategies against CRC.

## Author contributions

**Conceptualization:** Fangbin Zhou.

**Data curation:** Chang Zou.

**Formal analysis:** Wenyong Tan.

**Funding acquisition:** Yong Dai.

**Investigation:** Donge Tang, Liewen Lin.

**Resources:** Yong Dai.

**Supervision:** Yong Dai.

**Validation:** Fangbin Zhou.

**Writing – original draft:** Weier Dai.

**Writing – review & editing:** Fangbin Zhou.

## Supplementary Material

Supplemental Digital Content
