# Supplementary material for: Single-cell transcriptional profiling reveals the heterogenicity in colorectal cancer
Source: Medicine (Baltimore). 2019 Aug 23;98(34):e16916. doi: 10.1097/MD.0000000000016916 (PMC6716720; doi:10.1097/MD.0000000000016916)

**Supplemental Digital Contents**

Supplemental Digital Content 1. Violin plot indicating the expression distribution of the top 10 marker genes of cluster 2.


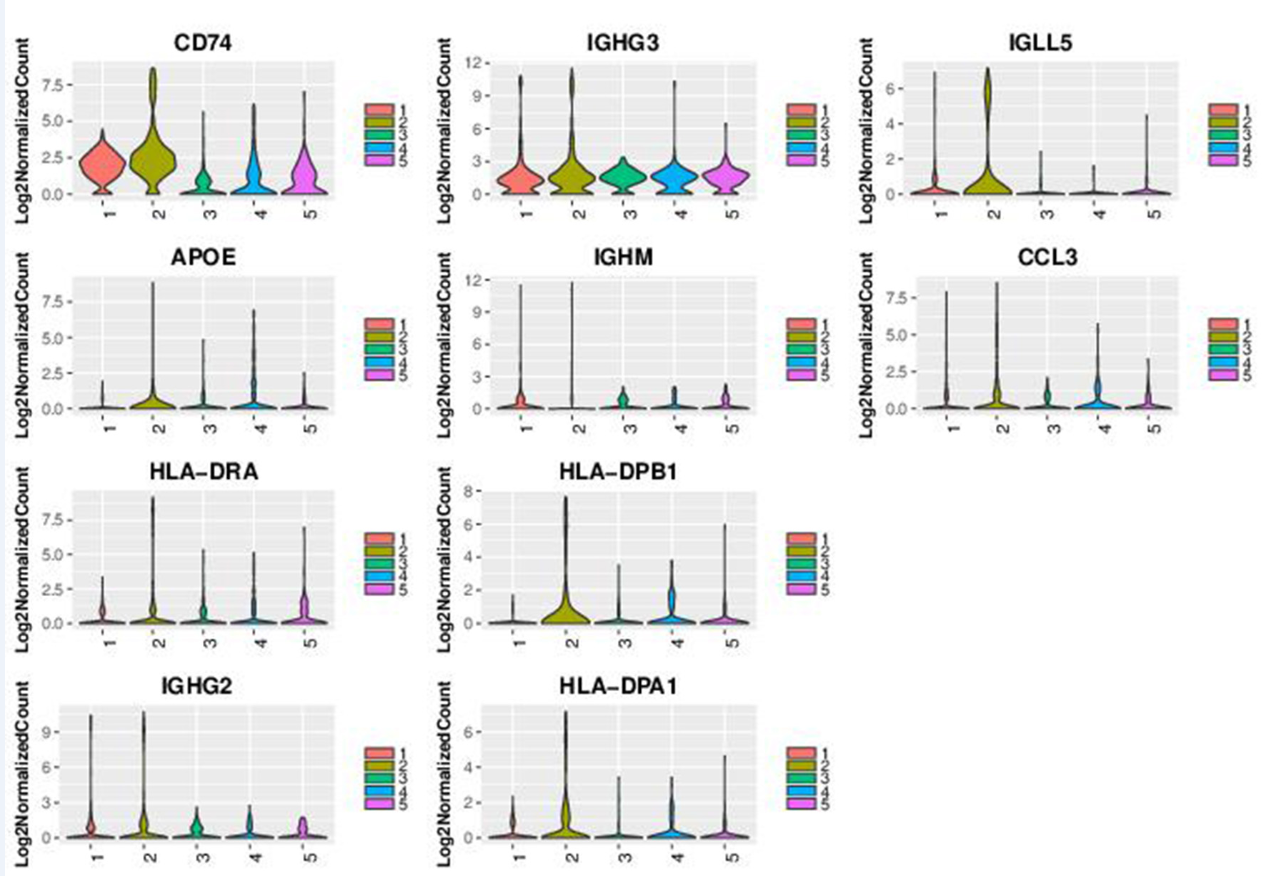


Supplemental Digital Content 2. Violin plot indicating the expression distribution of the top 10 marker gene of cluster 3.


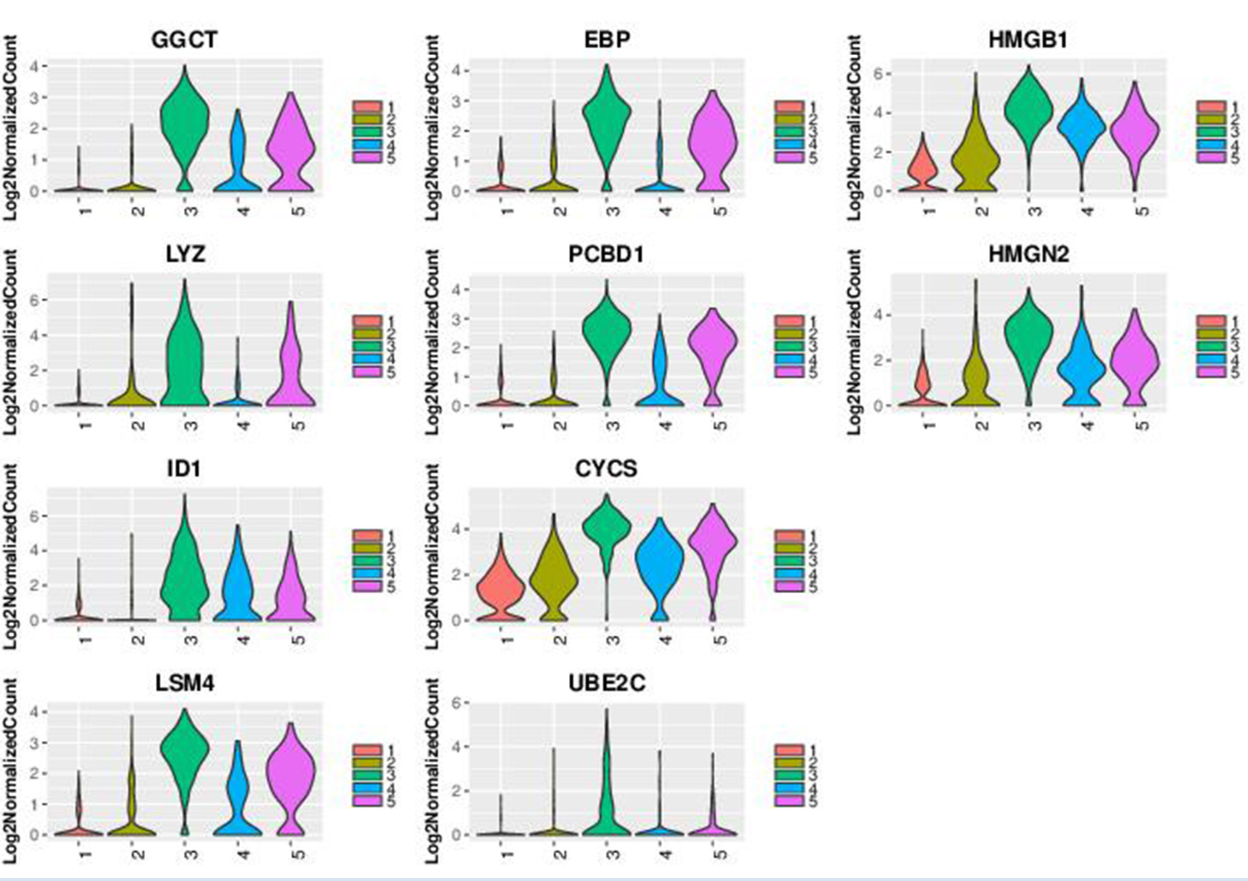


Supplemental Digital Content 3. Violin plot indicating the expression distribution of the top 10 marker gene of cluster 4.


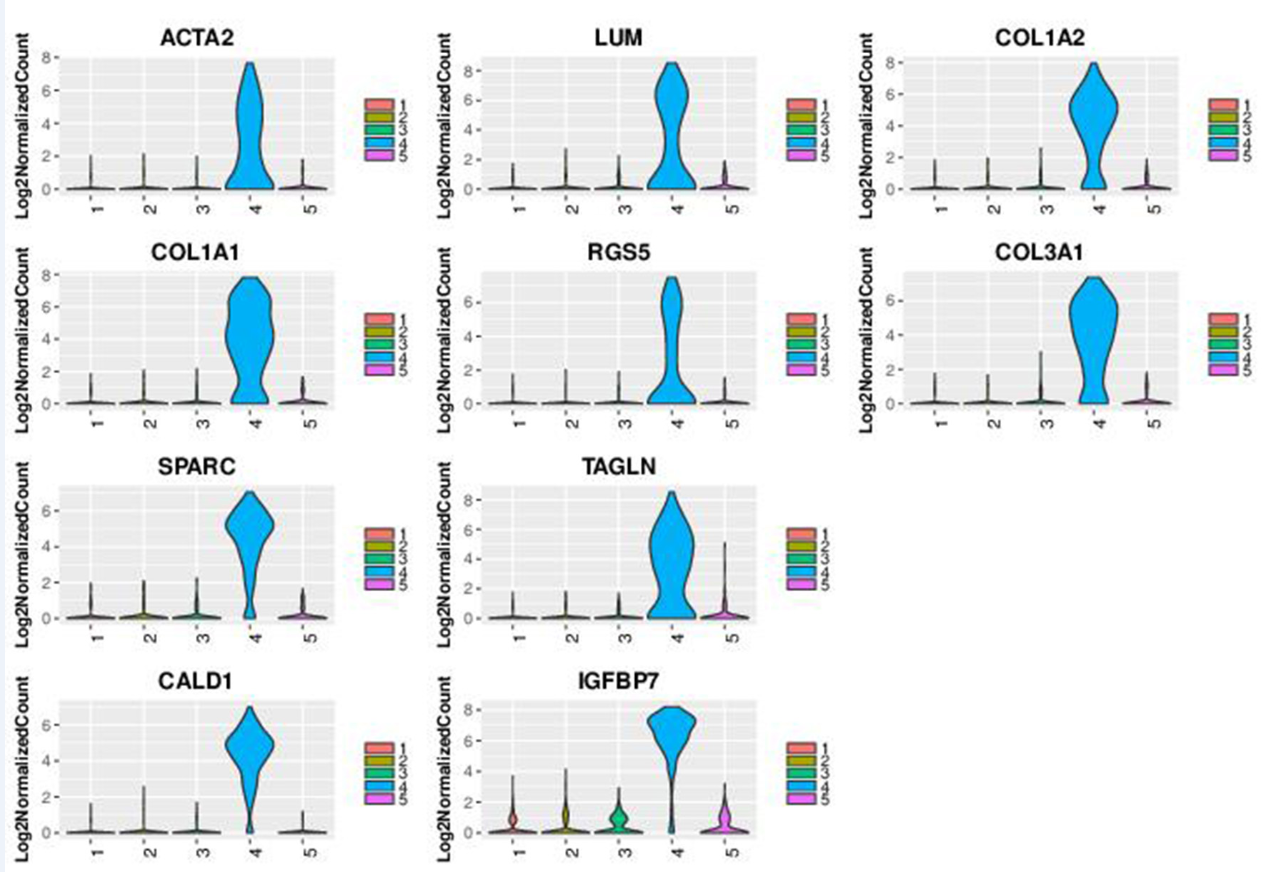


Supplemental Digital Content 4. Violin plot indicating the expression distribution of the top 10 marker gene of cluster 5.


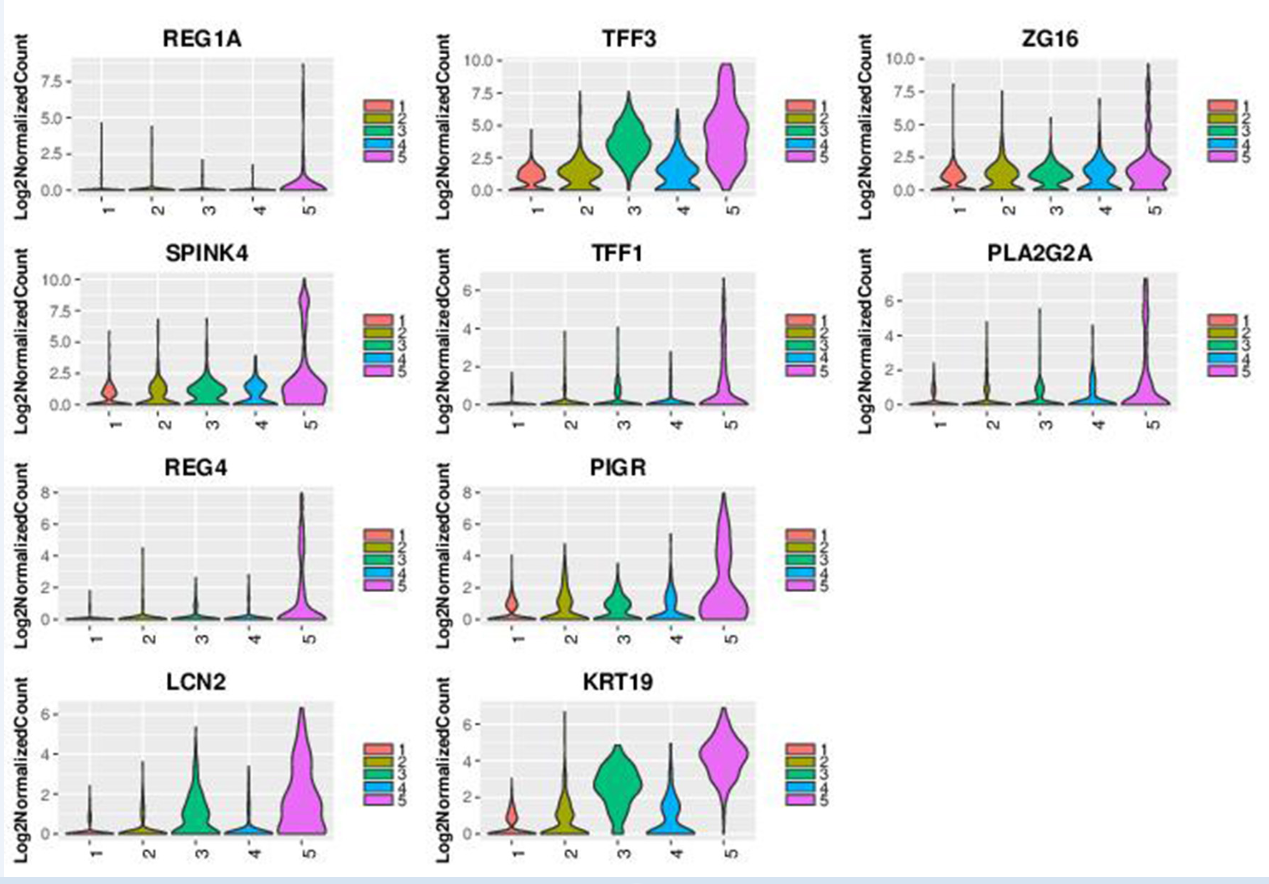


Supplemental Digital Content 5. (A) GO analysis of the selected top 10 GO terms for cluster 2; (B) Disease enrichment plot from KEGG pathway analysis for cluster 2.


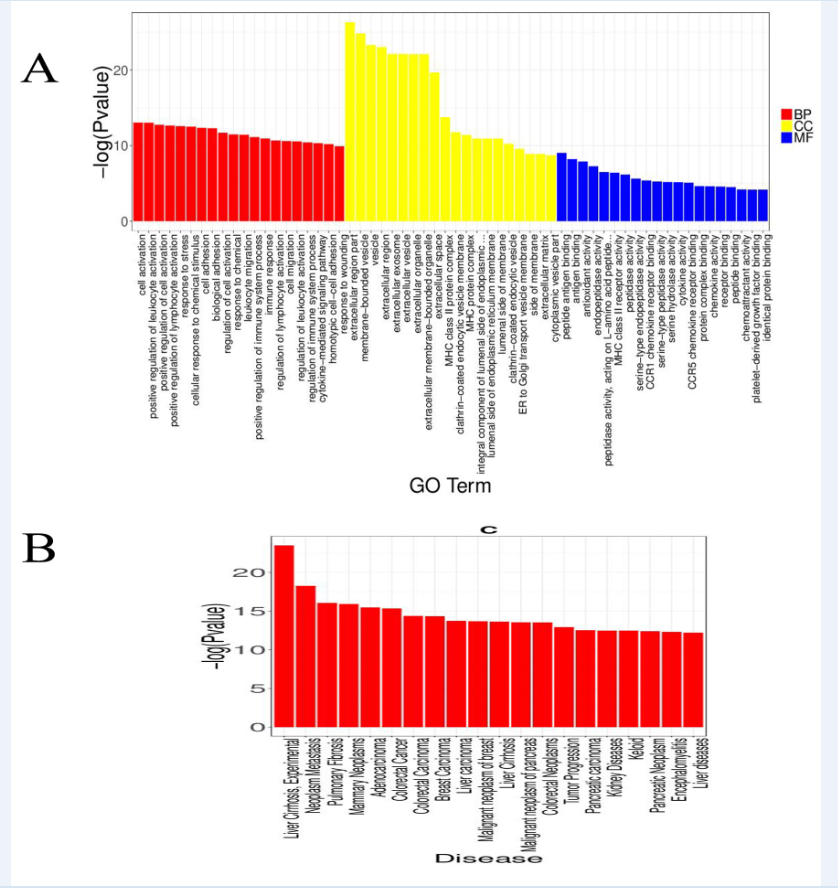


Supplemental Digital Content 6. (A) GO analysis of the selected top 10 GO terms for cluster 3; (B) Disease enrichment plot from KEGG pathway analysis for cluster 3.


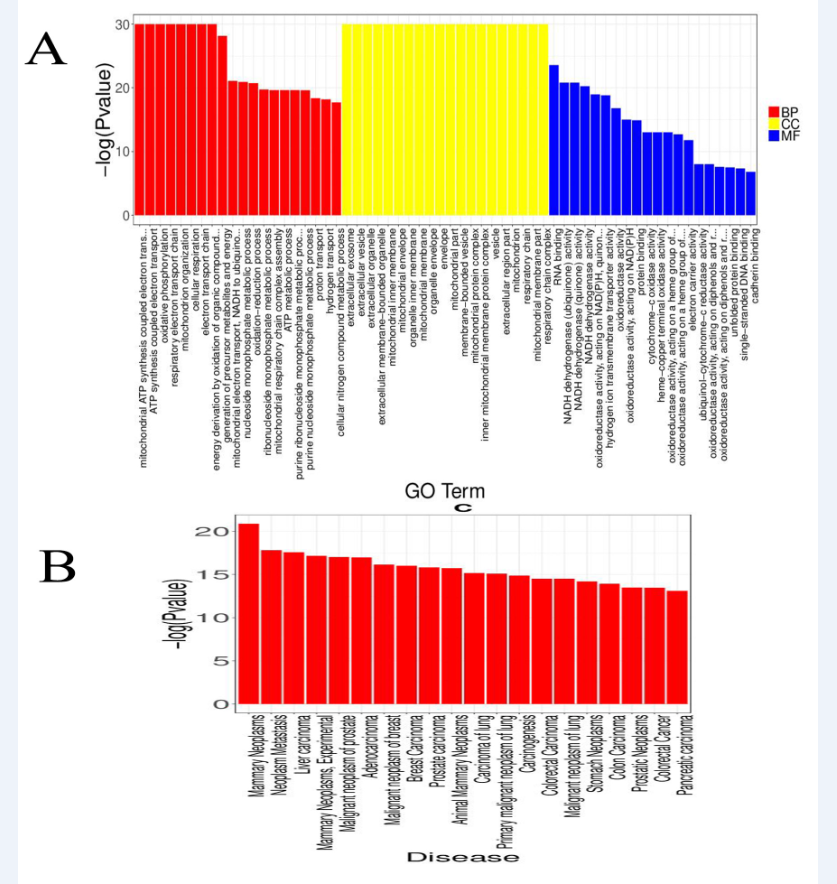


Supplemental Digital Content 7. (A) GO analysis of the selected top 10 GO terms for cluster 4; (B) Disease enrichment plot from KEGG pathway analysis for cluster 4.


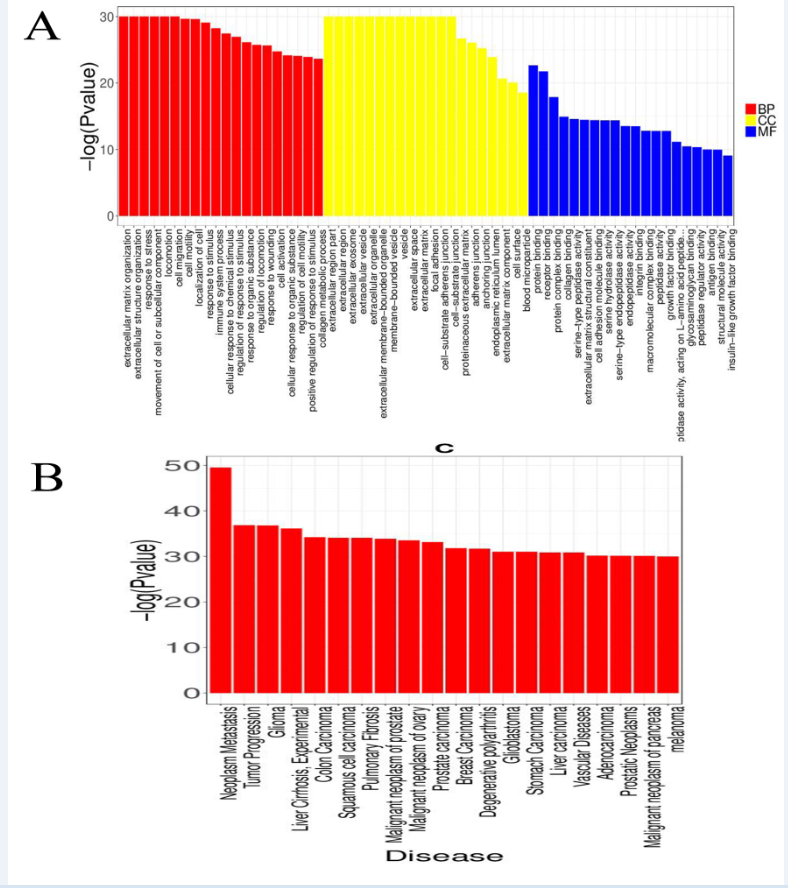


Supplemental Digital Content 8. (A) GO analysis of the selected top 10 GO terms for cluster 5; (B) Disease enrichment plot from KEGG pathway analysis for cluster 5.


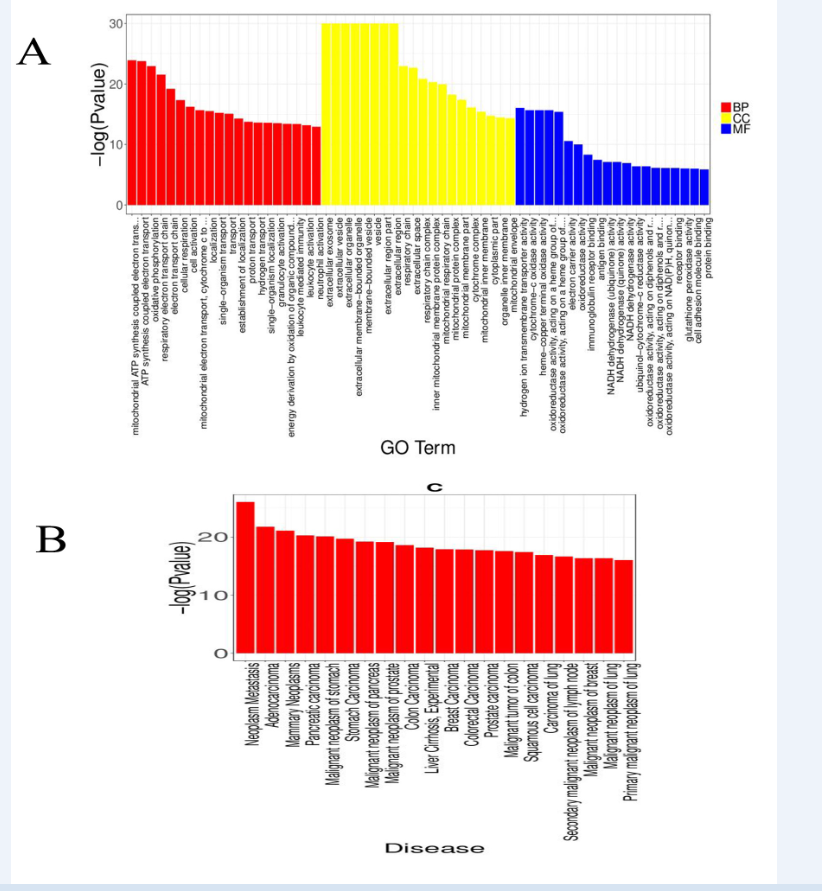

Supplement: Supplemental Digital Content [file medi-98-e16916-s001.doc]
